# The *fbpA/sapM* Double Knock Out Strain of *Mycobacterium tuberculosis* Is Highly Attenuated and Immunogenic in Macrophages

**DOI:** 10.1371/journal.pone.0036198

**Published:** 2012-05-04

**Authors:** Sankaralingam Saikolappan, Jaymie Estrella, Smitha J. Sasindran, Arshad Khan, Lisa Y. Armitige, Chinnaswamy Jagannath, Subramanian Dhandayuthapani

**Affiliations:** 1 Regional Academic Health Center and Department of Microbiology and Immunology, University of Texas Health Science Center at San Antonio, Edinburg, Texas, United States of America; 2 Department of Pathology and Laboratory Medicine, University of Texas Health Science Center at Houston, Houston, Texas, United States of America; Tulane University, United States of America

## Abstract

Tuberculosis (TB), caused by *Mycobacterium tuberculosis* (*Mtb*), is the leading cause of death due to bacterial infections in mankind, and BCG, an attenuated strain of *Mycobacterium bovis*, is an approved vaccine. BCG sequesters in immature phagosomes of antigen presenting cells (APCs), which do not fuse with lysosomes, leading to decreased antigen processing and reduced Th1 responses. However, an *Mtb* derived *ΔfbpA* attenuated mutant underwent limited phagosome maturation, enhanced immunogenicity and was as effective as BCG in protecting mice against TB. To facilitate phagosome maturation of Δ*fbpA*, we disrupted an additional gene *sapM*, which encodes for an acid phosphatase. Compared to the wild type *Mtb*, the Δ*fbpAΔsapM* (double knock out**;** DKO) strain was attenuated for growth in mouse macrophages and PMA activated human THP1 macrophages. Attenuation correlated with increased oxidants in macrophages in response to DKO infection and enhanced labeling of lysosomal markers (CD63 and rab7) on DKO phagosomes. An *in vitro* Antigen 85B peptide presentation assay was used to determine antigen presentation to T cells by APCs infected with DKO or other mycobacterial strains. This revealed that DKO infected APCs showed the strongest ability to present Ag85B to T cells (>2500 pgs/mL in 4 hrs) as compared to APCs infected with wild type *Mtb* or Δ*fbpA* or Δ*sapM* strain (<1000 pgs/mL in 4 hrs), indicating that DKO strain has enhanced immunogenicity than other strains. The ability of DKO to undergo lysosomal fusion and vacuolar acidification correlated with antigen presentation since bafilomycin, that inhibits acidification in APCs, reduced antigen presentation. Finally, the DKO vaccine elicited a better Th1 response in mice after subcutaneous vaccination than either Δ*fbpA* or Δ*sapM*. Since Δ*fbpA* has been used in mice as a candidate vaccine and the DKO (Δ*fbpA*Δ*sapM*) mutant is more immunogenic than Δ*fbpA*, we propose the DKO is a potential anti-tuberculosis vaccine.

## Introduction

Tuberculosis (TB), a major disease due to *Mycobacterium tuberculosis* (*Mtb*), kills about 1.8 million people and is the cause of latent infection in about a third of the human population. Control of tuberculosis has become more complicated due to multi-drug resistant (MDR) and extensively drug resistant (XDR) *Mtb* strains and AIDS. The attenuated *Mycobacterium bovis* Bacillus Calmette-Guérin (BCG) vaccine, administered for fifty years to over a billion people, does not protect against adult TB, while it affords variable protection against childhood TB and tuberculous meningitis [1], [2].

During the last decade, many novel vaccines have been tested in mouse or guinea pig models including DNA, subunit proteins, recombinant BCG and attenuated strains of *Mtb* [3], [4], [5]. Most DNA or subunit vaccines are based on immuno-dominant *Mtb* proteins like MPT32, Phos, DnaK, GroES, MPT46, MPT53, MPT63, 19 kDa lipoprotein, Antigen 85 (Ag85) complex (Ag 85A, Ag85B and Ag85C), RD1 encoded proteins like early secretory antigen target-6 (ESAT-6), culture filtrate protein 10 (CFP10), and antigen TB10.4 [5]. Some have been tested as combinations and others as fusion vaccines. Hybrid 1 (H-1) and HyVac-1 consisting of Ag85B-ESAT-6, and Ag85B-TB10.4 [6], [7] are some examples. Most DNA and subunit vaccines show efficacy more or less similar to BCG. However, recombinant BCG strains like BCG30 that overexpresses Ag85B [8], BCG::RD1-2F9 that has RD1 region integrated with chromosome to express ESAT-6 and related proteins [9] and rBCG: Δ*ureC*-*Hly* that expresses listeriolysin are more effective than BCG in animal models [10], [11]. Some of these are in phase I and II clinical trials [5], [12].

Since the RD1 region encoded major antigens ESAT-6 and CFP-10 are deleted in BCG, efforts have been made to examine whether attenuated mutants of wild type *Mtb* could serve as candidate vaccines. We reported first that the *fbpA* gene disrupted mutant (Δ*fbpA*) from wild type *Mtb* was attenuated for growth within macrophages [13] and was an effective vaccine in mice against tuberculosis [14]. Purine [15], leucine [16], proline/tryptophan [17] and lysine [18] auxotroph vaccines were described earlier as being attenuated in mice as well as protective against tuberculosis in mice. The pantothenate auxotroph vaccine followed these mutants [19], [20], [21], [22], [23], [24] and set the trend for multiple candidate vaccines derived from *Mtb*. The latter now include *Mtb fad26* [25], *mec-2/mec-3* [26], *RD1/panCD* [21], *phoP* [27], [28], [29], *19 kDa* [30], *sigE* [31] and *secA2/lysA* [32]. Despite inducing better protection in animal models, *Mtb* derived candidates are still far from human application due to safety concerns. More importantly, there seems to be a decreased understanding of the molecular basis of vaccine induced protection in comparing BCG vs. *Mtb* derived vaccines.

Protective Th1 immune response against TB depends on CD4 T cells secreting IFN-γ, IL-2 and TNF-α, and CD8 T cells secreting similar cytokines and producing perforin and granulysin [33], [34], [35]. CD4 and CD8 T cells are in turn, primed through MHC-II and MHC-I dependent pathways of peptide presentation by mycobacteria infected APCs. Furthermore, peptides are usually generated by lysosomal proteases, which mean that mycobacteria like *Mtb* or BCG vaccine need to be delivered into lysosomes for efficient peptide production. Paradoxically, it is a well-established fact that wild type *Mtb* and even BCG vaccine avoid phago-lysosomal fusion [36], [37], [38], [39], [40], [41].

It is becoming apparent that the inability of BCG vaccine to fuse with lysosomes affects its efficacy. Initially, Pancholi et al. found BCG growing in human monocytes sequestered from CD4 T cells [42]. We reported that the presence of BCG in near neutral pH phagosomes of macrophages leads to a reduced ability of macrophages to present the immune-dominant antigen 85B [43]. We also showed that Cathepsin-D was an important protease that produced Ag85B and was not activated in neutral pH of the phagosomes. Others reported that macrophages infected with recombinant BCG expressing Cathepsin-S protease were able to present Ag85B better since the novel BCG bypassed the need for lysosomal fusion [44]. Finally, we reported that enhanced delivery of BCG over-expressing Ag85B to lysosomes through autophagy increased antigen presentation *in vitro* and vaccine efficacy in mice [45]. Since Ag85B is a major component of anti-tuberculosis vaccines, and anti-tuberculosis vaccines need to be processed through the lysosomes to produce MHC-II dependent peptides, we have proposed the ability of APCs to present Ag85B as a good *in vitro* surrogate marker for vaccine efficacy.

In this context, very few attempts have been made to examine *Mtb* derived vaccines for their ability to undergo PL fusion. Our initial reports showed that *Mtb* derived attenuated Δ*fbpA* mutant that lacks Ag85A of the Ag85 complex is immunogenic in mice, partially phagosome maturation competent [13], [14], [46], protects against tuberculosis, and is capable of priming T cells more effectively than BCG. In this study, we examined the effect of deleting another gene in Δ*fbpA* mutant to render it more competent for PL fusion, presumably rendering it a better vaccine candidate. We hypothesized that deletion of *sapM* gene in *Mtb* Δ*fbpA* strain would further enhance the ability to undergo PL fusion. The gene *sapM* codes for an acid phosphatase [47], which plays a critical role during phagosome maturation by interfering with the levels of phosphotidylinositol 3-phosphate (PI3P) on the phagosomes [48]. PI3P is a lipid component required for docking of rab and rab effector proteins which regulate endosome trafficking and eventual acquisition of lysosomal constituents by phagosomes. *Mtb sapM* has been shown to hydrolyze (aka.dephosophorylation) PI3P to avoid maturation of *Mtb* containing phagosomes [48]. We demonstrate here that the Δ*fbpA*Δ*sapM* double knock out (DKO) mutant is not only more attenuated than Δ*fbpA,* but is also PL fusion competent and consequently, more immunogenic in macrophages and mice.

## Results

### Generation of Δ*fbpA*Δ*sapM* Double Knockout (DKO) Strain

**Figure 1:**
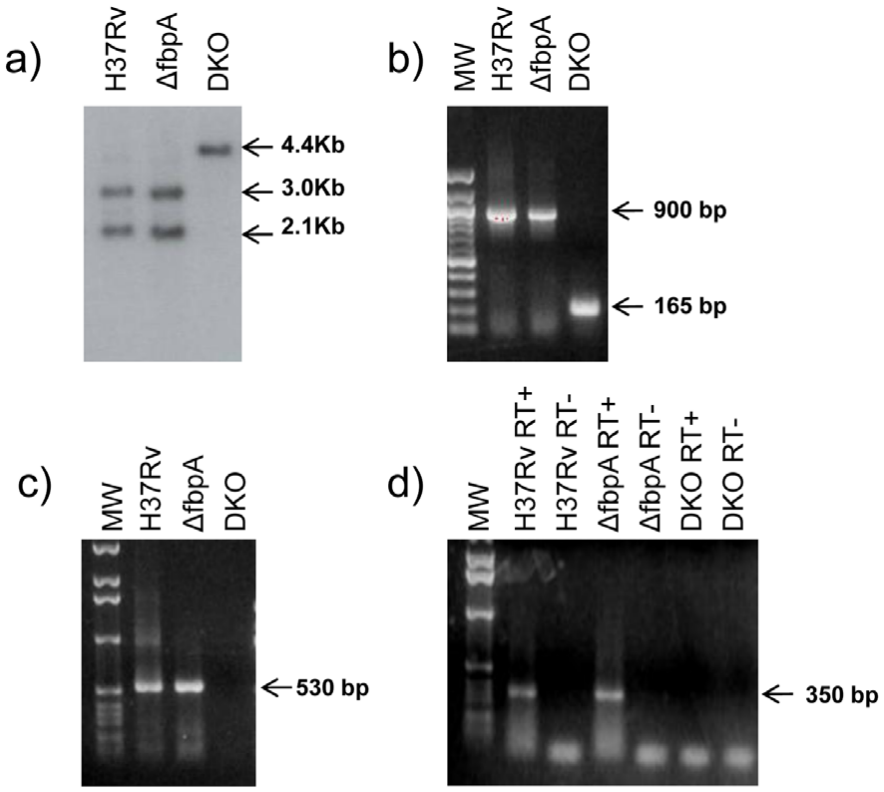
Southern and PCR analyses of *M. tuberculosis* strains. **a**). Southern analysis of genomic DNA of *M. tuberculosis (Mtb)* wild type (H37Rv), *fbpA* mutant (ΔfbpA) and *fbpA/sapM* double knock out (DKO) strains. Genomic DNA was digested with NdeI and BamHI, separated on 1% agarose gels and transferred to nitrocellulose membranes. Membranes were hybridized with [^32^P]dCTP labeled 3.3 kb DNA fragment containing *sapM* region and signals captured by autoradiography. Arrows indicate the sizes of the signals. **b**–**c**). PCR analysis for *sapM* region in *M. tuberculosis* H37Rv, ΔfbpA and DKO strains. PCR was performed using standard protocols with genomic DNA from the above strains as templates. Primer pairs RV3310EX1 and Rv3310EX2 (**b**) and RV3310EX1 and RV3310RT2 (**c**) were used to amplify DNA. **d**. RT-PCR analysis for *sapM* expression in *M. tuberculosis* H37Rv, ΔfbpA and DKO strains. Total RNA was used to synthesize cDNA from these strains. RT+ and RT- indicate cDNA templates generated in the presence or absence of reverse transcriptase (Superscript II; Invitrogen). The products obtained from both reactions were used as templates in RT-PCR to prove the absence of DNA contamination in total RNAs used for reverse transcriptions. PCR was performed using primers RV3310RT1 and RV3310RT2. MW: molecular weight marker; arrow indicates the size of the band. PCR products were separated on 1% agarose gels.

The creation of *Mtb* Δ*fbpA* strain and its characterization in macrophages and mice have already been described [13], [14], [46]. To generate an additional *sapM* gene deletion in Δ*fbpA*, the plasmid construct pTBSAPM5 was electroporated into this strain and cultures were plated initially on 7H10-TW-OADC agar with hygromycin and X-gal to obtain hygromycin resistant blue colonies. This selection resulted in several blue colonies out of which one colony designated as DKO/C was subjected to further screening to obtain sucrose resistant colonies lacking β-galactosidase activity. This screening resulted in three white colonies namely DKO/C1, DKO/C2 and DKO/C7. To verify if deletion of *sapM* gene had occurred in these colonies, a Southern blot was made with genomic DNA from these strains and also with *Mtb* H37Rv and *Mtb* Δ*fbpA*. Upon hybridization with 3.3 kb radiolabeled DNA probe containing *sapM* region, and subsequent autoradiography, the blot showed two signals (2.1 and 2.9 kb) for *Mtb* H37Rv and *Mtb* Δ*fbpA* strains and only one signal (4.4 kb) for DKO strains (DKO/C1 is shown here) (**Fig. 1a**). These signals were on the predicted line, based on restriction sites in this region of the genome (**Fig. S1**), and indicate that *sapM* gene is deleted by allelic replacement in DKO strain. To further confirm the deletion of *sapM* in DKO strain, we also performed PCR using primers specific for this region (please see **Fig. S1** for the location of the primers). While primers located at the 5′end (RV3310EX1) and 3′ end (RV3310EX2) of the *sapM* gene yielded the expected sizes of 900 bp and 165 bp DNA, respectively for the *Mtb* H37Rv and DKO strain (**Fig. 1b**), an internal primer RV3310RT2 with the primer at the 5′end of *sapM* (RV3310EX1) failed to amplify a 530 bp product in the DKO strain (**Fig. 1c**), again reinforcing the deletion of *sapM* gene in this strain. Finally, we determined whether the deletion of *sapM* gene in DKO strain led to the disruption of the expression of this gene by RT-PCR, using the internal primers RV3310RT1 and RV3310RT2. This revealed that only cDNA obtained from *Mtb* H37Rv and *Mtb* Δ*fbpA* strain only yielded the expected size DNA fragment (350 bp) but not the DKO strain (**Fig. 1d**), thus confirming the absence of *sapM* expression in DKO strain. This concluded the generation of *Mtb fbpA/sapM* double knockout (DKO) strain.

### Δ*fbpA*Δ*sapM* DKO Strain is Attenuated in Macrophages

**Figure 2:**
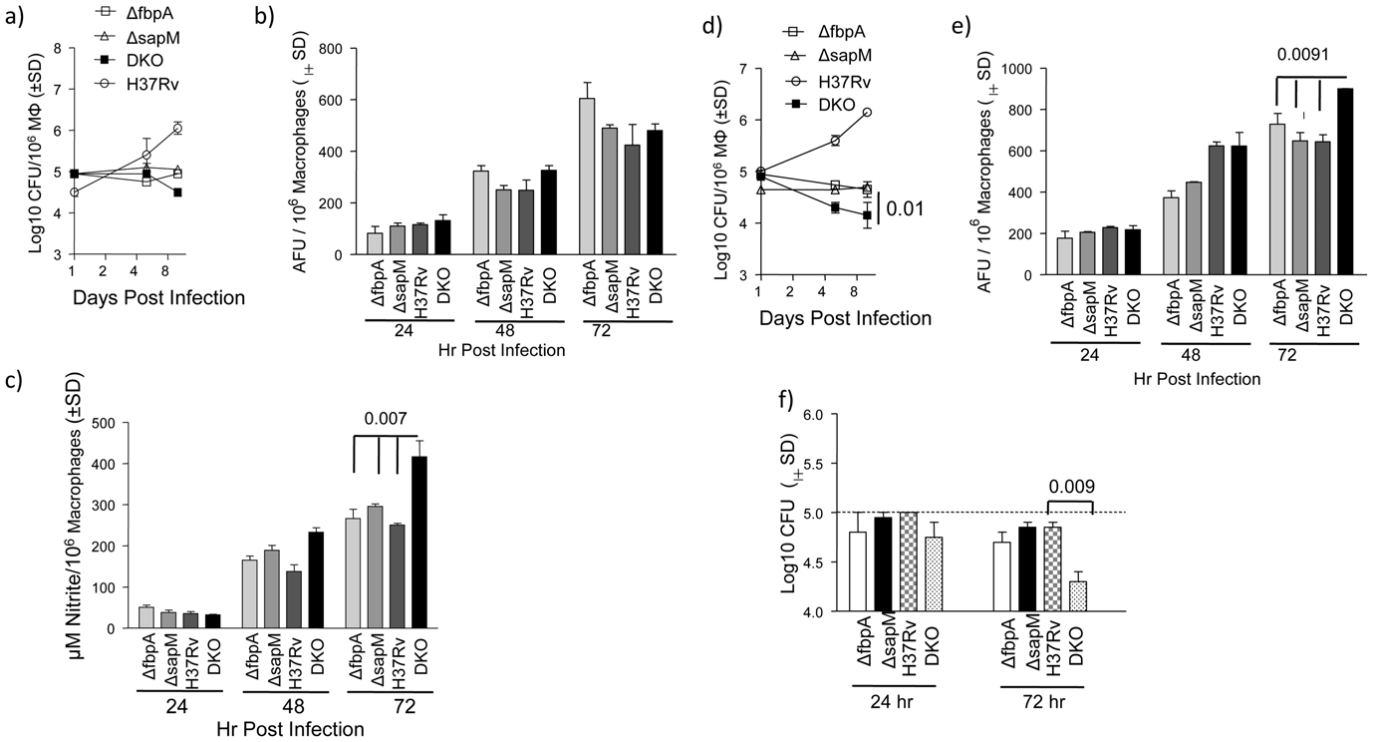
The Δ*fbpA*Δ*sapM* double knockout (DKO) strain is attenuated in macrophages and induces stronger oxidant responses that reduce its viability: Macrophages from C57Bl/6 mouse bone marrow (BMs) and human THP1 macrophages (pre-activated with phorbol ester) were infected with mycobacteria (MOI 1∶1), washed, incubated, lysed and plated for viable colony counts (CFUs). **a)**. The DKO strain is more attenuated compared to wild type *Mtb* in BMs. **b-c)** Intracellular reactive oxygen species (ROS) and nitric oxide (NO) were measured respectively using dihydro-dichloro-fluorescein acetate (DCFDA) fluorescent probe and Greiss reagent. DKO induced elevated NO responses (p value by t test; panel **c**) but not ROS (panel **b**). **d-e)** DKO was attenuated in THP1 macrophages compared to Δ*fbpA*, Δ*sapM* or wild type H37Rv in BMs (p<0.01) that correlated with increased ROS responses (panel **e**). Nitric oxide responses of THP1 were not detectable (not shown). **f)**. Mycobacteria (10^5^ CFU/mL; baseline shown as dotted line) were exposed to the bactericidal action of the superoxide and NO donor 3-morpholinosydnonimine (10 mM; SIN-1) in 7H9 broth and viable counts determined at intervals (24 and 72 hr post treatment) by plating on 7H11 agar. DKO is markedly susceptible by 72 hrs *in vitro* to the oxidants released by SIN-1 (p value by t test).

The ability of mycobacterial strains to grow inside macrophages is a virulence trait, and macrophages are routinely used to determine their virulence [14], [46]. **Fig. 2a & d** illustrate the growth of strains within mouse bone-marrow derived macrophages (BMs) and human THP1 macrophages. The DKO strain was relatively attenuated in both BMs and THP1 macrophages, compared to Δ*fbpA* and Δ*sapM* mutants or the wild type *Mtb* H37Rv (**Fig. 2a,d**). To determine if the enhanced attenuation of DKO was due to *sapM*, we complemented the DKO mutant strain with *sapM* gene and named the strain as DKOcom. BMs infected with the DKOcom showed a growth curve similar to that of its parental strain Δ*fbpA* (**Fig. S2**), indicating that the increased attenuation of DKO was due to deletion of *sapM*.

Since, the intracellular death of mycobacteria is partly due to oxidative radicals like reactive oxygen species (ROS) and nitric oxide (NO), macrophages and culture supernatants were evaluated respectively for ROS using a fluorescent probe and NO derived nitrite with Griess reagent. The BMs showed no significant differences in ROS responses but a marginally elevated NO response was induced by the DKO strain (**Fig. 2b,c**). In contrast, the ROS response was elevated for THP1 macrophages infected with DKO compared to other strains (**Fig. 2e**). There was no significant NO response observed among THP1 macrophages infected with either wild type or mutants (not shown). This observation was consistent with the notion that human macrophages produce barely detectable NO during mycobacterial infection [**49**].

ROS cascade begins with the generation of superoxide by phagocyte oxidase [43]. Superoxide and inducible nitric oxide synthase derived NO have bacteriostatic and bactericidal activity, respectively against mycobacteria. To confirm the susceptibility of DKO to oxidants, the superoxide and NO donor, 3-morpholinosydnonimine (SIN-1) was used to treat a highly viable culture of wild type and mutants in broth culture. **Fig. 2f** shows that DKO strain was again more susceptible to oxidants compared to others. These data suggest that the decreased growth of DKO was attributable in part to elevated oxidant responses in macrophages. Similar studies were done using macrophages infected with DKO, although it was difficult to rule out artifacts arising due to the dose-dependent toxic effects of oxidants on macrophages.

### Δ*fbpA*Δ*sapM* DKO Strain is Processed Efficiently Through Phago-lysosomal Fusion

**Figure 3:**
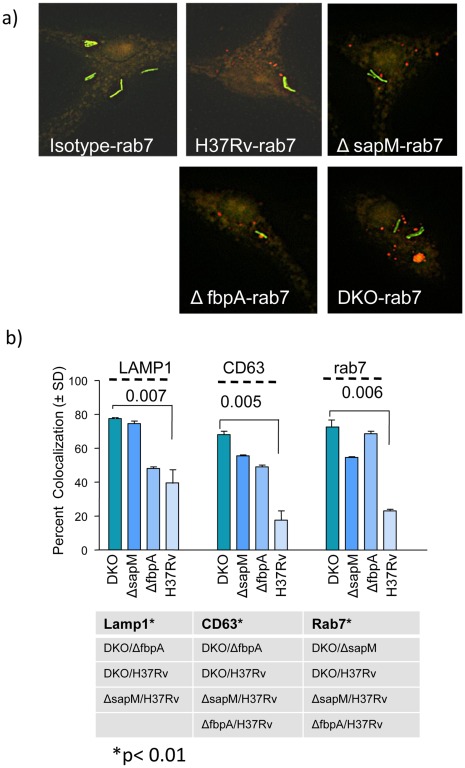
The Δ*fbpA*Δ*sapM* double knockout (DKO) strain shows enhanced lysosomal localization in mouse macrophages: *gfpMtb* H37Rv or Oregon green stained mutant strains were phagocytosed into BMs, incubated, fixed 24 hrs later and stained with primary antibodies to lysosomal markers LAMP1 (IDB4), CD63 and rab7 followed by Texas red conjugated conjugates. Mycobacteria colocalizing with antibodies were scored using a Nikon fluorescence microscope and Metaview deconvolution software. **a**) Illustration that the DKO mutant colocalizes better with rab7 lysosomal marker. **b**) Percent colocalization was determined by counting 200 macrophages per well each with 1–3 mycobacteria and averaging counts from triplicate chambers (SD). One of three similar experiments is shown. Text below the bar diagram indicates the colocalization of each marker in relation to different strains (*p<0.01, t test).

Although *Mtb* H37Rv resists phago-lysosomal fusion [37], [41], [50], certain mutant *Mtb* strains have a decreased ability to prevent PL fusion, and this decreased ability correlates with reduced intracellular viability for these mutants [46], [51]. Since lysosomes present an acidified hostile environment, and the parent Δ*fbpA* mutant was partially PL fusion competent, we reasoned that PL fusion is one additional mechanism through which, intracellular viability of DKO strain could be reduced. To test this hypothesis, BMs and THP1 macrophages were infected with either GFP tagged *Mtb* wild type (H37Rv) or Oregon green stained mutants (Δ*fbpA,* Δ*sapM and* DKO).The PL fusion was monitored using microscopic colocalization of lysosomal markers like CD63 and rab7 (**Fig. 3a**). An antibody to lysosome associated membrane protein-1 (LAMP-1), was used as a positive control since LAMP-1 is present on all mycobacterial phagosomes but at varying levels between virulent and avirulent bacteria [46]. The DKO strain extensively colocalized with LAMP-1 followed by *ΔsapM, ΔfbpA* and H37Rv (Table, **Fig. 3b**). Significantly, CD63 and rab7 were found to be more enriched on DKO phagosomes compared to either *ΔsapM* or *ΔfbpA* or H37Rv. Since CD63 and rab7 are definitive markers of lysosomes, and LAMP1 is present both on late endosomes and lysosomes, these data indicated that DKO phagosomes are lysosome fusion competent. It was also significant to note that *ΔsapM* and *ΔfbpA* stained for CD63 and rab7 more densely than wild type H37Rv, indicating that deletion of either *fbpA* or *sapM* renders these mutants comparatively more lysosome fusion competent than wild type H37Rv (**Fig. 3b**).

### Δ*fbpA*Δ*sapM* DKO Strain is More Immunogenic in Macrophages and in Mice

**Figure 4:**
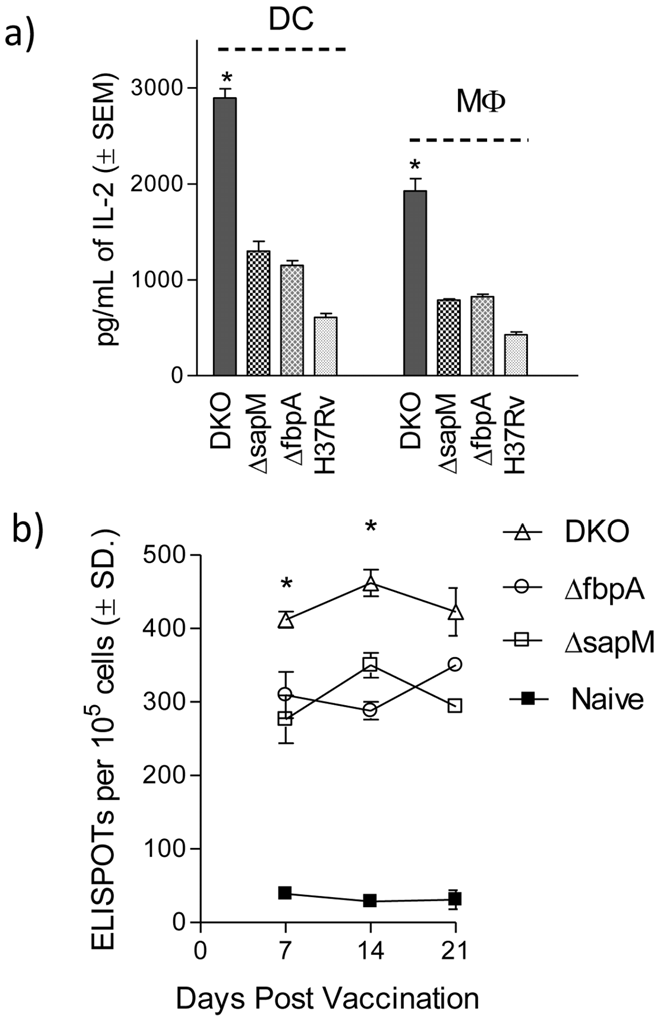
The Δ*fbpA*Δ*sapM* double knockout (DKO) strain is more immunogenic in mouse macrophages and elicits stronger immune responses in mice: **a**) BMs and DCs from C57Bl/6 mice were infected with mycobacteria (MOI 1∶1), washed and overlaid with Antigen 85B specific BB7 hybridoma T cells (1∶20 ratio). After 4 hrs, the supernatants collected were tested for IL-2 using sandwich ELISA. DKO induces BMs and DCs to prime T cells to secrete larger amounts of IL-2, indicating a better processing of DKO for Ag85B (4 experiments, SEM, * <0.009 vs. **Δ**fbpA *or* **Δ**sapM; by t test). **b**). C57Bl/6 mice (3 per group) were vaccinated with mycobacterial strains at 10^6^ CFU per mouse given once subcutaneously. At time intervals, the spleen derived T cells were tested for Ag85B responsive T cells using IFN-γ coated plates and Elispot assay. DKO vaccination leads to a larger expansion of Ag85B specific T cells. All Elispot numbers represent Ag85B stimulated numbers subtracted from KLH protein stimulated T cells. T cells from naïve mice were stimulated with KLH alone (3 separate experiments, 3 mice per group per time point).

Since DKO strain showed increased PL fusion and enhanced susceptibility to killing within macrophages, we hypothesized that DKO could be more immunogenic since PL fusion leads to degradation of mycobacterial antigens facilitating their presentation through the MHC-II pathway. When mycobacteria infect macrophages, an Ag85B derived peptide-25 epitope is rapidly presented to T cells [52]. We demonstrated that, *in vitro* presentation of Ag85B is a measure of immunogenicity of mycobacteria in macrophages [43] and PL fusion of mycobacteria within macrophages enhances Ag85B production predicting vaccine efficacy against tuberculosis [45]. *In vitro* antigen presentation using dendritic cells and macrophages, indicated that the DKO strain was more efficiently processed since overlaid T cells secreted more IL-2 (>2500 pgs/mL in 4 hrs) than either Δ*sapM* (1000 pgs/mL) or Δ*fbpA* (950 pgs/mL) infected APCs (**Fig. 4a**).

Finally, *in vitro* immunogenicity was correlated with the ability of DKO to prime Th1 immunity *in vivo*. Mice were vaccinated subcutaneously with 10^6^ CFU of mutants and analyzed for Ag85B specific T cells using Elispot. The DKO strain again induced an increase in the number of Ag85B specific T cells in mouse spleens (**Fig. 4b** **).** These data indicate that the DKO (Δ*fbpA*Δ*sapM*) has a highly immunogenic phenotype in macrophages and DCs as well as in mice. It may be noted that the numbers of spot forming cells (SFU) increased over 14 days followed by a decline. This is consistent with the splenic immune response following mycobacterial vaccines. For example, SFUs for Ag85B increase after a single vaccination with BCG over 2 weeks, and then decline [45].

## Discussion

The use of BCG as a vaccine against tuberculosis is mainly due to its genetic identity (over 90%) with *Mtb*, including genes encoding immunodominant antigens. However, comparison of genome sequences of *M. bovis* BCG with that of *Mtb* in late 90s raised several new issues. It was noticed that *M. bovis*, the parent strain of BCG, lacked approximately 120 ORFs in comparison with *Mtb* genome, which includes sequences that code for some regulatory proteins [53], [54]. In addition, BCG lacked the RD1 region that encodes the major immunogens ESAT-6 and CFP-10 [55], [56]. These differences indirectly implied that the antigenic profiles of BCG vaccine would vary considerably from *Mtb,* and BCG might not fully protect against tuberculosis. Attenuated mutants from wild type *Mtb* were therefore generated to be used as vaccines against tuberculosis [23]. Although the attenuated *Mtb* mutants reported so far have been found to be variably effective against experimental tuberculosis, some candidates have shown better efficacy against tuberculosis in mice compared to BCG vaccine. The selection of these vaccines was based primarily on the growth attenuation within macrophages [21], [23], [24], although some induced better levels of IFN-γ secreting T cells in mice compared to BCG. Nevertheless, there are no striking differences in modulation of host immune system by these vaccines and the parameters of *Mtb*-derived vaccine induced immunogenicity continue to be unclear. We sought to develop a novel DKO strain that is both attenuated and immunogenic.

The criteria for defining immunogenicity of a vaccine have remained diffuse and a surrogate marker has not been available to predict vaccine efficacy. Our initial studies proposed an *in vitro* Ag85B presentation as a surrogate marker to predict the immunogenicity of genetically altered BCG vaccine strains and mutants from wild type *Mtb*. Initially described by Harding’s group, this assay depends on the rapid processing and presentation of the immuno-dominant Ag85B by APCs to T cells that secrete IL-2 upon antigen recognition. This assay has also been used by others to monitor antigen processing within APCs [57]. We found that the ability of mycobacterial vaccines to produce Ag85B epitope within APCs generally correlated with the ability to protect against tuberculosis in mice [43], [44], [45], [58]. In this study, we therefore used the ability of APCs to process Ag85B as a marker for determining the immunogenicity of DKO. This seemed to be a valid approach since MHC-II-bound peptides drive CD4-dependent anti-tuberculosis TH1 response, and CD4 T cell deficient human HIV-1 infected patients rapidly succumb due to tuberculosis [59].

We propose that the increased processing of DKO in macrophages that led to enhanced *in vitro* antigen presentation, was due to an increased delivery of DKO to the lysosomes, which in turn, was due to the double-gene deletions. In our earlier studies, we found that Δ*fbpA* phagosomes fused readily with late endosomes but still avoided lysosomes [46]. This was presumably due to the phosphatase like activity of *fbpA* enzyme (unpublished observations). By deleting *sapM* encoded phosphatase, we enhanced the ability of DKO to bypass maturation inhibition and DKO showed enhanced lysosomal localization in macrophages (**Fig. 3**). Thus, our intended plan to disrupt *sapM* and alter trafficking of DKO succeeded.

It should be noted however that, a previous attempt to disrupt *sapM,* only marginally affected the phenotype of BCG vaccine [60]. In this regard, we propose following explanations. First, *sapM* presumably needs to be released out of the phagosomes to cause dephosphorylation of PI3P, and BCG phagosome membrane is not known to be easily permeable. Cloning of listeriolysin into BCG for example, enhances antigen release from phagosomes of BCG and better CD8 T cell responses [10]. Secondly, we disrupted *sapM* within Δ*fbpA* which is known to mature better than wild type *Mtb* H37Rv and deletion of *sapM* had a complementing effect on its ability to fuse with lysosomes [46]. On the other hand, reconstitution of *sapM* increased the growth within macrophages of the complemented DKOcom strain.

We therefore propose a novel concept that, the immunogenicity of mycobacterial vaccine candidates can be enhanced by rational deletion of mycobacterial genes that adversely regulate phagosome traffic. This approach is in contrast with earlier approaches, where the immunogenicity of candidate vaccines was enhanced by adding more genes through recombinant DNA technology [8], [9], [10].

Enhanced delivery to lysosomes and the apparent susceptibility of DKO to oxidants rendered the mutant more attenuated for growth in macrophages. This may be a benefit since candidate vaccines need to be safer in animal models and humans. In summary, our study suggests that *Mtb* derived candidates developed with emphasis on enhanced antigen processing are more likely to be useful vaccines against tuberculosis, and DKO mutant appears to be a potential vaccine candidate.

## Materials and Methods

### Ethics Statement

This study was carried out in strict accordance with the recommendations of the Guide for the Care and Use of Laboratory Animals of the National Institute of Health. The protocol was approved by the Institutional Animal care and Use Committee of the University of Texas Health Science Center, Houston (Protocol number AWC-09-175 under Animal Welfare Assurance Number A3413-01).

### Bacterial Strains, Media, and Growth Conditions

All bacterial strains, unless specified, were grown at 37°C. *Escherichia coli* strain DH5α was used to subclone *M. tuberculosis* DNA. LB broth or LB agar plates were used to grow *E. coli*. The above media with the antibiotic ampicillin (100 µg/mL) or kanamycin (25 µg/mL) or hygromycin (100 µg/mL) were used to grow *E. coli* strains containing plasmids. *Mtb* H37Rv (27294) is from ATCC. *M. tuberculosis* Δ*fbpA* strain is a derivative of H37Rv, which we published earlier [13]. *M. tuberculosis* Δ*sapM* strain was from NIH TB resources Center at Colorado State University (currently BEI, ATCC). Middlebrook 7H9 broth medium containing OADC (10%) and Tween (0.05%) (7H9 broth), or Middlebrook 7H10 agar medium containing OADC (10%) and Tween (0.05%) (7H10 Agar) or Middlebrook 7H11 agar medium containing OADC (10%) (7H11 Agar) was used to grow *M. tuberculosis* strains. Kanamycin (25 µg/mL) or hygromycin (50 µg/mL) was added to the Middlebrook media to grow *M. tuberculosis* strains harboring plasmids.

### Disruption Plasmid for *M. tuberculosis sapM*

To disrupt *sapM* (*Rv3310*) gene in *Mtb* Δ*fbpA* strain, we first downloaded the DNA sequences of *sapM* gene and its adjacent region from NCBI databases. Based on the sequences, we synthesized four oligonucleotide primers namely RV3310A, RV3310B, RV3310C and RV3310D (Table 1). Synthesis of oligonucleotide primers were performed at the Center for DNA Technology, University of Texas Health Science Center at San Antonio. While the primers RV3310A, RV3310B were designed to amplify the 5′ prime region of *sapM* and its upstream 1306 bp fragment (Frag I), primers RV3310C and RV3310D were designed to amplify the 3′ region of *sapM* and its downstream 934 bp fragment (Frag II). Also primers Rv3310B and RV3310C were engineered to have StuI sites in them. Using these primers and *Mtb* H37Rv DNA, we amplified fragments I and II in PCR and these fragments were cloned into pCR2.1 Vector (Invitrogen) to create plasmids pTBSAPM1 and pTBSAPM2, respectively. The fragment II from plasmid pTBSAPM2 was released by cutting with StuI and BamHI and cloned into the pTBSAPM1 cut with the same restriction enzymes. The resulting plasmid pTBSAPM3 has a DNA fragment, which has upstream and downstream regions of *sapM* but with substantial deletion in *sapM* coding region (**Fig. S1**). The DNA fragment in pTBSAPM3 was released from the plasmid by digesting the plasmid with restriction enzymes HindIII and NotI and the released fragment cloned into p1NIL to create plasmid pTBSAPM4. A 7939 bp PacI fragment, that contains *sacB* and *lacZ* genes and a gene for hygromycin resistance, was then isolated from the plasmid pGOAL19 [61] and cloned into the PacI site of the plasmid pTBSAPM4. The resulting plasmid pTBSAPM5 was used to generate a markerless *sapM* mutant in *Mtb* Δ*fbpA* strain. Plasmid DNA from *E. coli* was isolated by using a Qiaperp kit (Qiagen Inc.,Valencia, Calif.).

**Table 1:** Oligonucleotide primers used in this study.

Primer	Primer sequence (5′––3′)
Rv3310A	TGGTGTACGCCTACGAGGAA
Rv3310B	TAGGCCTAGCAACGATGCTGCCAGGAC
Rv3310C	GAGGCCTCTACAACGTGCTGTCCACAT
Rv3310D	TCGGTCGATCATCCAGGTAA
Rv3310EX1	CATGAGGATCCCATGCTCCGCGGAATCCAGGCTC
Rv3310EX2	CGAGGATCCCTAGTCGCCCCAAATATCGGTTATTGG
Rv3310RT1	GTCATCGTGGTGGAGGAGAA
Rv3310RT2	GTCGTCGGCACGTTACTGAA

### Electroporation and Screening for the Δ*fbpA*Δ*sapM* Mutant

To obtain Δ*fbpA*Δ*sapM* double mutant, we used Δ*fbpA* strain reported earlier [13]. This mutant strain was grown to mid-logarithmic phase (OD_600_ = 0.8–1.0) in 7H9 broth and competent cells prepared according to Jacobs et al. [62]. Four hundred microliter of Δ*fbp* cells were mixed with 5 µg pTBSAPM5 DNA, linearized with NaOH treatment, in 0.2 mm cuvettes (BioRad) and electroporated using standard protocols. After electroporation, 1 mL 7H9 medium without any antibiotic was added to each cuvette and left overnight at 37°C. Then, the cell suspension from the cuvettes was plated on 7H10 agar plates containing the antibiotic hygromycin (50 µg/mL) and X-gal (40 µg/mL). Transformants showing blue color, resulting from single crossover event, were selected after three weeks of incubation at 37°C. Further screening of the transformants for the deletion of *sapM* region was performed by a two-step selection method [61]. First, the blue colonies were streaked onto 7H10 agar plates containing no antibiotics. Following growth, a loop-full of cells were resuspended into liquid medium, diluted serially to several folds and plated onto 7H10- agar plates containing 2% sucrose. Sucrose resistant colonies resulting from double crossover event were streaked onto plates with or without hygromycin. Colonies showing no resistance to hygromycin were finally streaked onto plates containing kanamycin, since *fbpA* mutant is kanamycin resistant. The DNA from these colonies were further examined in Southern and PCR to confirm the deletion of *sapM* region.

### Complementation of DKO Strain

To complement the DKO strain with *sapM* gene, we constructed an integration plasmid carrying *sapM* gene as follows. First, we amplified the whole *sapM* gene and its upstream promoter region (2116 bp) by PCR using primers RV3310A and RV3310EX2 (Table 1) and *Mtb* H37Rv genomic DNA. The fragment was cloned in pCR2.1 vector to result in plasmid pTBSAPMA. This plasmid was digested with KpnI and XbaI to release the fragment which was cloned in KpnI and XbaI digested pMV306H, a derivative of pMV306 in which kanamycin resistant marker is replaced with hygromycin marker, to get plasmid pM306SAPMA. This plasmid was transformed into DKO/C1 (Δ*fbpA/*Δ*sapM* double mutant) strain by electroporation. The colonies were selected in 7H10 hygromycin plates and the integration of the plasmid was confirmed by Southern blot (data not shown). The resulting strain was named as DKOcom.

### Southern and PCR Analysis

To confirm the deletion of *sapM* gene in *Mtb ΔfbpA* strain, we performed Southern analysis [63]. We isolated chromosomal DNA from *Mtb* H37Rv, *Mtb* Δ*fbpA and Mtb* Δ*fbpA*Δ*sapM* strains using CTAB (cetyltrimethylammonium bromide) method [64] and 3 µg of each DNA was digested with NdeI and BamHI. The digested fragments were separated on 1% agarose gel and Southern transferred to nitrocellulose membrane (BioRad). The blot was hybridized with a [α-^32^P]dCTP labeled probe generated by random primer method using the 3300 bp DNA fragment (generated by primers Rv3310A and Rv3310D) as template. Southern hybridization and final washing of the blot were performed at 68°C. In addition to Southern, we also carried out PCR to confirm the deletion of *sapM* region in *Mtb* Δ*fbpA*Δ*sapM* strain using standard protocol [63] with Taq polymerase (Perkin-Elmer, Foster City, Calif.). We used primers Rv3310EX1, Rv3310EX2 and Rv3310RT2 (Table 1) for this analysis.

### RNA Isolation and RT-PCR

Total RNA from *Mtb* strains was isolated using Tri reagent as described previously [65]. cDNA from total RNA was synthesized using Superscript (Invitrogen) and random hexamers. PCR analysis was performed with primers RV3310RT1 and RV3310RT2 (Table 1) and using the cDNA from the previous step as the template for the reaction.

### Macrophages and T Cells

Bone marrows from C57BL/6 (4–8 weeks) mice were cultured for 7–10 days in Iscove's modification of Dulbecco's modified Eagles medium (IDMEM) with 10% FBS and 10 ng/mL GM-CSF (Cell Sciences, USA). The macrophages (BMs) and dendritic cells (DCs) were purified using CD11c bead fractionation kit (Miltenyi Inc, USA) yielding >95% pure DCs and >95% adherent BMs (effluent of bead fractionation) [46]. They were plated onto 24 well plates (for colony counts, oxidant measurement) or 8-well slide chambers (for antibody stains) and were rested in IDMEM without GM-CSF before infections. In addition to mouse derived cells, phorbol mystyl acetate (PMA) (10 nM) activated human THP1 cells were used to test the intracellular growth of mycobacteria. The T cell hybridoma (BB7) cell line specific for an epitope (241–256) of Ag85B of *Mtb* was used in antigen presentation assays (kindly provided by Dr. C. Harding, Case Western University, Cleveland, OH). The culture of these cell lines were described earlier [43], [46].

### Determination of Intracellular Growth or Survival of *Mtb* Strains

To determine the growth curves in macrophages, the wild type and mutants were used to infect (MOI of 10) naïve BMs and PMA activated THP1 cells using procedures described earlier [14], [43], [46]. Phagocytosis was allowed to take place for 4 h, after which the monolayers were washed to remove the non-phagocytosed bacteria. One mL of fresh IDMEM medium was added to each well and the culture plates were incubated at 37°C in the presence of 5% CO_2_. After each time point, macrophages were harvested and lysed with 0.05% sodium dodecyl sulfate (SDS) for 15 min at room temperature. Tenfold dilutions of the lysed macrophage suspensions were made in PBS and 100 µL of each dilution was examined for *Mtb* growth on triplicate 7H11 agar plates that were incubated for 3 weeks at 37°C. Each *Mtb* strain was examined in quadruplicate wells and three independent experiments were performed.

### Oxidant Assay and Oxidant Susceptibility of Mycobacteria in Macrophages

BMs and THP1 macrophages infected with mycobacteria were tested for ROS using the fluorescent probe H_2_-dichlorodihydrofluorescein diacetate (H_2_DCFDA)(Invitrogen, USA) that is cleaved within the cells by esterases and oxidized into fluorescent DCF reactive oxygen species (ROS) induced by infection [43]. Fluorescent DCF was measured by reading BMs or THPs of 24 well plates using in Ascent fluoroscan at 485 nm/530 nm. Triplicate wells of macrophages were read for each mutant at different time intervals in two separate experiments and plotted as average fluorescence units (AFU). The enzyme inducible nitric oxide synthase generated nitric oxide (NO) was measured in the supernatants of similar cultures using Griess reagent and expressed as µM nitrite in the medium. The susceptibility of mycobacteria to superoxide and NO released by the donor 3-morpholinosydnonimine N-ethylcarbamide (SIN-1)(Invitrogen, USA) was determined by incubating 10^5^ CFU/mL of mycobacteria in a broth culture with 10 mM of SIN-1 for 24 and 72 hrs and plating organisms on 7H11 agar for viable counts.

### Phago-lysosomal Localization of *Mtb* Strains

Macrophages were infected with GFP expressing *Mtb* H37Rv and Oregon green stained Δ*fbpA*, Δ*sapM* and DKO (Δ*fbpA*Δ*sapM*) strains. These strains were sonicated slightly to disperse the bacteria, centrifuged at 500 rpm and the supernatant containing the single CFUs were used for infection. BMs of slide chambers were infected with *Mtb* strains (MOI, 1) for 4 h at 37°C in the presence of 5% CO_2,_ washed, and incubated with fresh medium up to 72 h. Washing, fixing of the cells, staining for different markers and mounting of the slides were performed as reported earlier [66]. Texas red conjugated antibodies to primary antibodies were from Jackson Immunochemicals (West Grove, PA). Colocalization was examined and scored using a Nikon fluorescence microscope equipped with a Metaview deconvolution software as described [67].

### *In vitro* Antigen 85B Presentation Assay

Monolayers of BMs were infected with *Mtb* strains for 4 hrs, (MOI 1∶5) washed and overlaid with Ag85B specific BB7 T cells (1∶20 ratio) as described earlier [43]. Four or 18 hrs later the supernatant was assayed for IL-2 using a sandwich ELISA kit (R and D systems, CA) and expressed as pg/mL/10^6^ T cells. This assay has been validated earlier by the lack of antigen presentation when macrophages are infected with *Mtb* strains deleted for Ag85B antigen (Δ*fbpB*) or when MHC class II deficient PMJ2-R macrophages are used as antigen presenting cells [43]. Two or four separate experiments were performed for each *Mtb* strain using triplicate wells for each assay and the data were averaged for *p* value calculations.

### Elispot Assay

C57Bl/6 mice (3 per group per time point per strain) were immunized s.c. with 10^6^ CFU of *Mtb* strains once and after 7,14 and 21 days, the splenic cells were depleted of non-T cells using a pan-T cell kit (Miltenyi Inc). A 96 well plate coated with anti-mouse IFNγ (Elispot kit, Ebiosciences) was washed and overlaid with 10^5^ enriched T cells from spleens of mice along with T cells from naïve mice. The wells were added with 100 ng of Ag85B purified protein per well (BEI,ATCC). Control wells received 100 ng/mL of KLH protein and Elispots from this control were subtracted from those induced by Ag85B restimulation and expressed as spot forming cells per 10^5^ T cells. Three separate experiments were performed and data averaged.

## Supporting Information

Figure S1 **Schematic showing the restriction sites and primers around** ***sapM*** **region.** a). *sapM* region in the genome of Wild type (H37Rv) *M. tuberculosis.* Stippled box represents the *sapM* gene; empty boxes on either side represent the flanking regions; BamHI, EcoRI, NdeI, NotI and PstI are restriction enzymes around *sapM* gene. Numbers above the small black boxes indicate the location of the different primers to amplify the DNA or cDNA. 1, RV3310A; 2, RV3310B; 3, RV3310C; 4, RV3310D; 5, RV3310EX1; 6, RV3310EX2; 7, RV3310RT1; 8, RV3310RT2. Lines below the boxes indicate the sizes of DNA fragments obtained when cut with BamHI and NdeI. b). PCR fragments amplified from the *sapM* region. FragI was amplified by primers RV3310A and RV3310B and FragII was amplified by primers Rv3310C and RV3310D. Stippled boxes represent the *sapM* gene; empty boxes on either side represent the flanking regions. C). The *sapM* region cloned into plasmid pTBSAPM5. Stippled box represents the *sapM* gene; empty boxes on either side represent the flanking regions. D. *SapM* region in the chromosome of DKO (*fbpA/sapM* double knock out) strain. Stippled box represents the *sapM* gene; empty boxes on either side represent the flanking regions BamHI, EcoRI, NotI and PstI are restriction enzymes around *sapM* gene. Line below the boxes represent the size of the DNA fragment obtained when cut with BamHI.
(TIF)

Figure S2 **Viability of DKO strain complemented with** ***sapM*** **gene (DKOcom) in bone marrow derived macrophages.** Macrophages from C57Bl/6 mouse derived bone marrow (BMs) were infected with mycobacteria (MOI 1∶1), washed, incubated, lysed and plated for viable colony counts (CFUs). Results indicate that DKOcom strain shows a growth pattern similar to that of its parental strain *ΔfbpA*, which is higher than that of DKO strain (P≤0.001).
(TIF)
