# Supplementary figures and images for: The fbpA/sapM Double Knock Out Strain of Mycobacterium tuberculosis Is Highly Attenuated and Immunogenic in Macrophages
Source: PLoS One. 2012 May 4;7(5):e36198. doi: 10.1371/journal.pone.0036198 (PMC3344844; doi:10.1371/journal.pone.0036198)

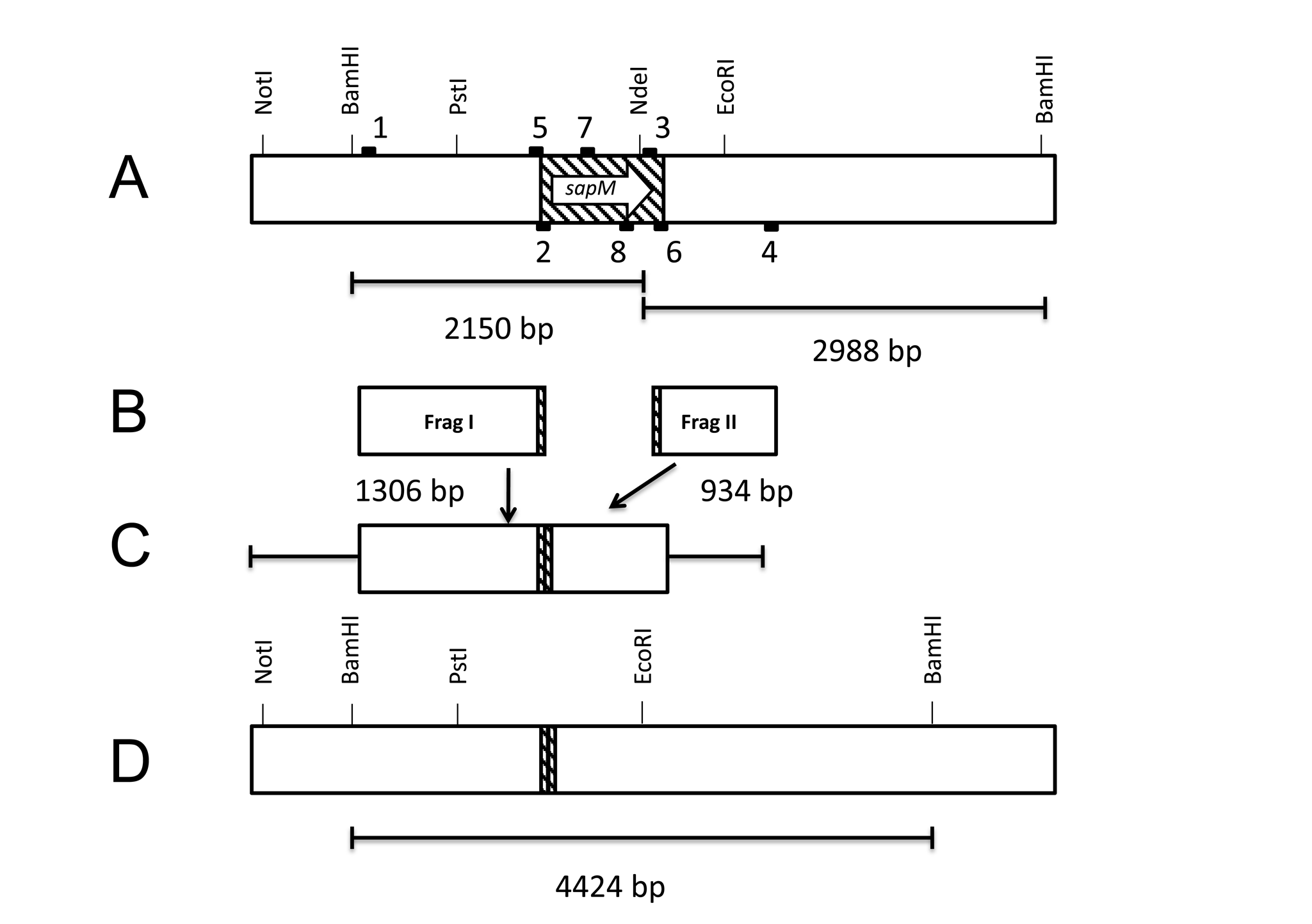

Supplement: Figure S1 — Schematic showing the restriction sites and primers around sapM region. a). sapM region in the genome of Wild type (H37Rv) M. tuberculosis. Stippled box represents the sapM gene; empty boxes on either side represent the flanking regions; BamHI, EcoRI, NdeI, NotI and PstI are restriction enzymes around sapM gene. Numbers above the small black boxes indicate the location of the different primers to amplify the DNA or cDNA. 1, RV3310A; 2, RV3310B; 3, RV3310C; 4, RV3310D; 5, RV3310EX1; 6, RV3310EX2; 7, RV3310RT1; 8, RV3310RT2. Lines below the boxes indicate the sizes of DNA fragments obtained when cut with BamHI and NdeI. b). PCR fragments amplified from the sapM region. FragI was amplified by primers RV3310A and RV3310B and FragII was amplified by primers Rv3310C and RV3310D. Stippled boxes represent the sapM gene; empty boxes on either side represent the flanking regions. C). The sapM region cloned into plasmid pTBSAPM5. Stippled box represents the sapM gene; empty boxes on either side represent the flanking regions. D. SapM region in the chromosome of DKO (fbpA/sapM double knock out) strain. Stippled box represents the sapM gene; empty boxes on either side represent the flanking regions BamHI, EcoRI, NotI and PstI are restriction enzymes around sapM gene. Line below the boxes represent the size of the DNA fragment obtained when cut with BamHI. (TIF) [file pone.0036198.s001.tif]

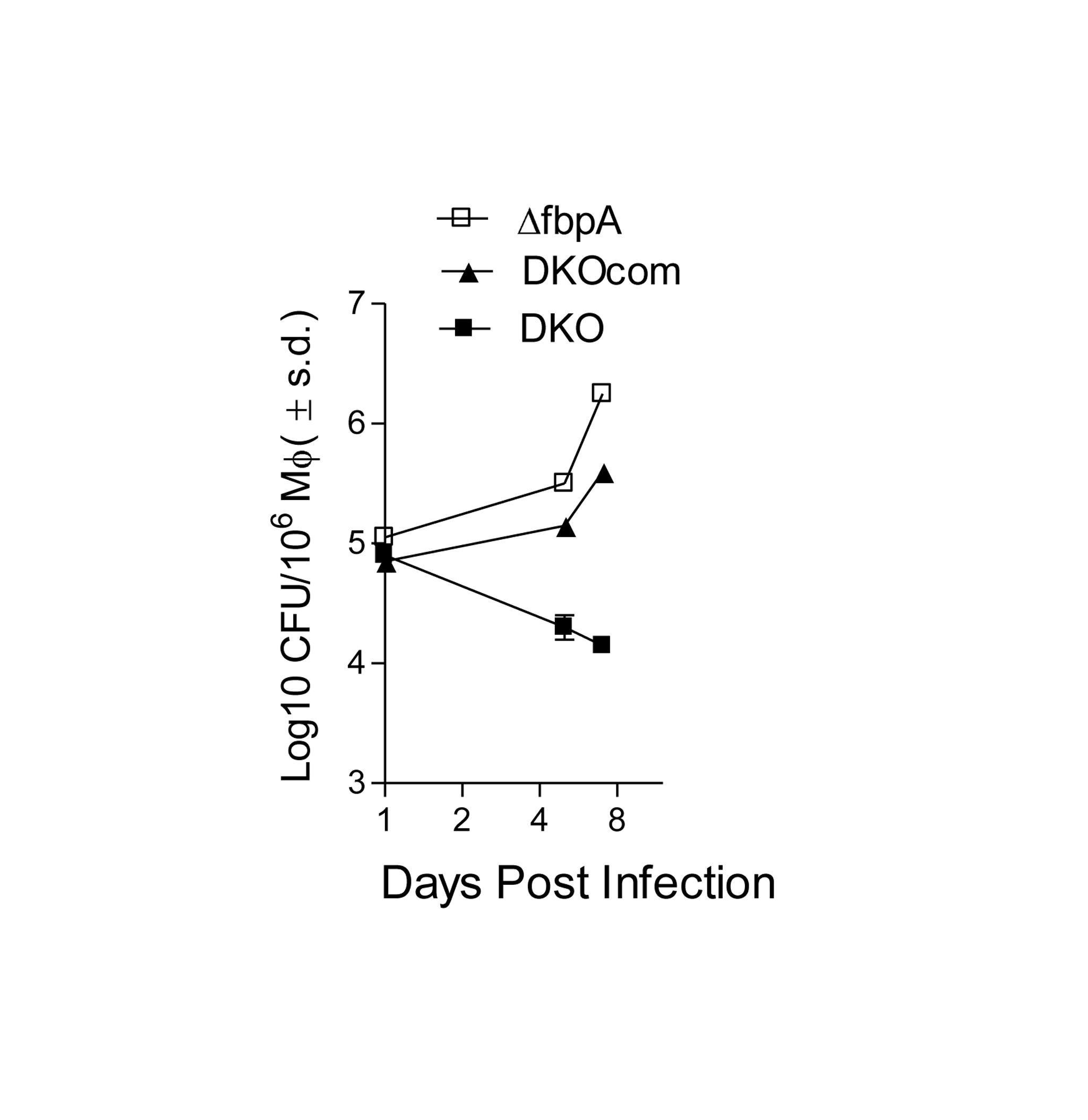

Supplement: Figure S2 — Viability of DKO strain complemented with sapM gene (DKOcom) in bone marrow derived macrophages. Macrophages from C57Bl/6 mouse derived bone marrow (BMs) were infected with mycobacteria (MOI 1∶1), washed, incubated, lysed and plated for viable colony counts (CFUs). Results indicate that DKOcom strain shows a growth pattern similar to that of its parental strain ΔfbpA, which is higher than that of DKO strain (P≤0.001). (TIF) [file pone.0036198.s002.tif]
